# Protective effects of galangin against H_2_O_2_/UVB-induced dermal fibroblast collagen degradation via hsa-microRNA-4535-mediated TGFβ/Smad signaling

**DOI:** 10.18632/aging.203750

**Published:** 2021-12-10

**Authors:** Jian-Jr Lee, Shang-Chuan Ng, Yean-Tin Ni, Jian-Sheng Liu, Chih-Jung Chen, Viswanadha Vijaya Padma, Chih-Yang Huang, Wei-Wen Kuo

**Affiliations:** 1 Department of Plastic and Reconstructive Surgery, China Medical University Hospital, Taichung 40447, Taiwan, ROC; 2 School of Medicine, China Medical University, Taichung 40447, Taiwan, ROC; 3 Department of Biological Science and Technology, College of Life Sciences, China Medical University, Taichung 406, Taiwan, ROC; 4 Ph.D. Program for Biotechnology Industry, China Medical University, Taichung 406, Taiwan, ROC; 5 China Medical University Beigang Hospital Thoracic Department, Yunlin 651, Taiwan, ROC; 6 Division of Breast Surgery, Department of Surgery, China Medical University Hospital, Taichung 40447, Taiwan, ROC; 7 Translational Research Laboratory, Department of Biotechnology, School of Biotechnology and Genetic Engineering, Bharathiar University, Coimbatore 641046, Tamil Nadu, India; 8 Cardiovascular and Mitochondrial Related Disease Research Center, Hualien Tzu Chi Hospital, Buddhist Tzu Chi Medical Foundation, Hualien 970, Taiwan, ROC; 9 Center of General Education, Buddhist Tzu Chi Medical Foundation, Tzu Chi University of Science and Technology, Hualien 970, Taiwan, ROC; 10 Department of Medical Research, China Medical University Hospital, China Medical University, Taichung 404, Taiwan, ROC; 11 Graduate Institute of Biomedical Sciences, China Medical University, Taichung 404, Taiwan, ROC; 12 Department of Medical Laboratory Science and Biotechnology, Asia University, Taichung 413, Taiwan, ROC

**Keywords:** galangin, H_2_O_2_, hsa-miR-4535, Smad4, UVB

## Abstract

This study aimed to investigate the mechanism underlying the protective effects of galangin against H_2_O_2_/UVB-induced damage using *in vitro* and *in vivo* models of photodamage. Moreover, we identified the involvement of miRNA regulation in this process. The H_2_O_2_/UVB-treated HS68 human dermal fibroblasts and UVB-induced C57BL/6J nude mice were used as *in vitro* and *in vivo* models of photodamage. The results showed that galangin treatment alleviated H_2_O_2_/UVB-induced reduction in cell viability, TGFβ/Smad signaling impairment, and dermal aging. Based on the results of microRNA array analyses and database searches, hsa-miR-4535 was identified as a potential candidate miRNA that targets Smad4. *In vitro*, galangin treatment activated Smad2/3/4 complex and inhibited hsa-miR-4535 expression in H2O2/UVB-exposed cells. *In vivo*, topical application of low (12 mg/kg) and high doses (24 mg/kg) of galangin to the dorsal skin of C57BL/6J nude mice significantly alleviated UVB-induced skin photodamage by promoting TGFβ/Smad collagen synthesis signaling, reducing epidermal hyperplasia, wrinkle formation, and skin senescence, as well as inhibiting hsa-miR-4535 expression. Taken together, our findings indicate a link between hsa-miR-4535 and TGFβ/Smad collagen synthesis signaling and suggest these factors to be involved in the photo-protective mechanism of galangin in dermal fibroblasts against H_2_O_2_/UVB-induced aging. The evidence indicated that galangin with anti-aging properties can be considered as a supplement in skin care products.

## INTRODUCTION

The clinical signs of human skin aging include increased wrinkling, laxity, and irregular pigmentation [1]. The process of skin aging is complicated and can be divided into two types: intrinsic aging and extrinsic aging. Intrinsic aging is due to the passage of time and genetic factors, while extrinsic aging, also called photoaging, mainly results from exposure to ultraviolet radiation [2, 3]. Previous studies have shown that abundant reactive oxygen species (ROS) are generated during both intrinsic and extrinsic aging. The accumulation of ROS can induce mitogen-activated protein kinase (MAPK) signaling and activate its downstream transcription factor, activator protein-1 (AP-1). Activated AP-1 can translocate to nucleus and bind to the promoter regions of matrix metalloproteinases (MMPs) [4]. MMPs are a large zinc-dependent endopeptidase group, contributing to the degradation of skin dermis extracellular matrix (ECM). Particularly, MMP-1, a collagenase, is involved in the cleavage of collagen types I and III [5]. Collagen types I and III, the major components of ECM, are capable of conferring support and strength to the skin dermis and their disarrangement leads to the characteristic wrinkling and laxity of aged skin [6].

Transforming growth factor beta (TGFβ) is a ubiquitous and potent cytokine with three different isoforms, TGFβ1, TGFβ2, and TGFβ3, that can positively regulate collagen synthesis in human skin dermis [2]. TGFβs initiate their signal by interacting with specific cell surface serine/threonine kinase receptor complexes, including TGFβ receptor types I (TβRI) and II (TβRII). TβRI phosphorylation triggers the phosphorylation of R-Smads (Smad2 and Smad3). Activated R-Smads interact with Smad4 to regulate its nucleus translocation and transcriptionally activate collagen types I and III through binding to promoters of Smad-binding elements (SBE) [7, 8]. Mounting evidence indicates that elevated ROS generation, induced by intrinsic or photoaging, contributes to TGFβ/Smad signaling pathway impairment, which in turn results in reduced collagen synthesis in human skin dermal fibroblasts [9, 10].

Galangin (3,5,7-trihydroxyflavone), a natural member of the flavonoid family, is abundant in *Alpinia officinarum* and propolis. Galangin is a potential candidate for treating ischemic stroke, diabetes, and different cancer types [11–13]. In addition, galangin possesses radical scavenging and anti-inflammatory activities [14, 15]. We previously showed that galangin reduced H_2_O_2_-induced inflammation and promoted collagen formation through insulin-like growth factor 1 receptor (IGFI-R)/ERK1/2 signaling pathways in HS68 cells [16, 17]. Furthermore, a study indicated that galangin was identified as compound of *Alpinia officinarum* and possesses collagenase inhibitory effects in dermal fibroblast cells [18]. However, a more detail molecular mechanism related to how galangin regulates dermal skin aging is unknown.

MicroRNAs (miRNAs) are a group of small, single-stranded, non-coding RNA molecules with an average length of 22 nucleotides. Mature miRNAs are transcribed from DNA sequences. In most cases, mature miRNAs bind to the 3′-untranslated regions (UTRs) of target mRNAs to silence mRNA translation [19]. In human skin, miRNAs play crucial roles in the regulation of cell metabolism, cancer, senescence and collagen formation [20–23]. For example, miR-377 can induce dermal fibroblast senescence through inhibiting DNA methyltransferase 1 (DNMT1) expression, which subsequently leads to less methylation in p53 promoter and dermal fibroblast senescence [22]. The miRNA profile in UVB-induced damage in human dermal papilla cells (nHDPs) was previously investigated [24]. However, how UVB-induced dermal fibroblast senescence through miRNA regulation, especially in the involvement of natural compound is largely unknown. This study aimed to investigate the protective effects of galangin against H_2_O_2_/UVB-induced dermal damage as well as the role of microRNA-mediated collagen degradation in this process. We further aimed to identify the microRNAs involved in regulating collagen synthesis.

## RESULTS

### Galangin inhibits UVB/H_2_O_2_-induced cytotoxicity in HS68 human dermal fibroblasts

**Figure 1:**
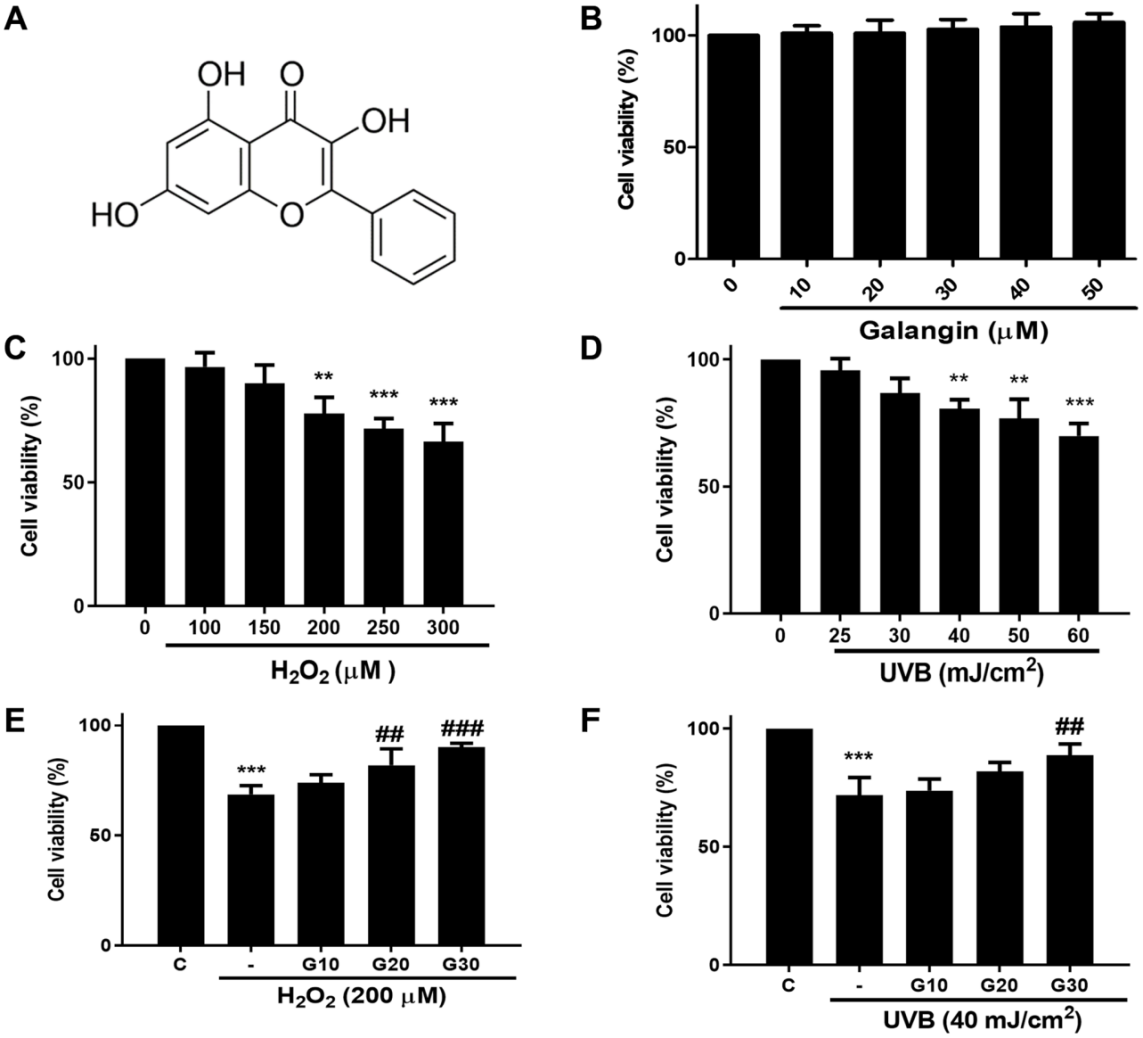
**Galangin inhibits UVB/H_2_O_2_-induced cytotoxicity in HS68 human dermal fibroblasts.** (**A**) The chemical structure of galangin. (**B**) Cell viability of HS68 cells treated with different concentrations of galangin (10, 20, 30, 40, 50 μM) for 24 h. (**C** and **D**) Cell viability of HS68 cells exposed to different doses of H_2_O_2_ (100, 150, 200, 250, 300 μM) or irradiated with UVB (25, 30, 40, 50, 60 J/cm^2^) for 24 h. (**E** and **F**) Cell viability of HS68 cells exposed to H_2_O_2_ (200 μM) or irradiated with UVB (40 J/cm^2^) for 1 h and then treated with galangin (10, 20, 30 μM) for 23 h. The cytoprotective effects of galangin were determined by the MTT assay. Control cells were assigned 100% viability. Values are shown as mean ± SE. Quantification of the results is shown (*n* = 3) ^*^P < 0.05, ^**^P < 0.01, ^***^P < 0.001 vs. untreated control cells; ^##^*P* < 0.01, ^###^*P* < 0.001 vs. H_2_O_2_ or UVB-treated cells.

To investigate the cytotoxicity of galangin (Figure 1A) *in vitro*, HS68 cells were treated with different dosses of galangin (0, 10, 20, 30, 40, 50 μM) for 24 h. MTT assay results showed that galangin was not cytotoxic to HS68 cells (Figure 1B). Next, we exposed HS68 cells to different doses of H_2_O_2_ (0, 100, 150, 200, 250, 300 μM) and UVB (0, 25, 30, 40, 50, 60 mJ/cm²) for 24 h. H_2_O_2_ and UVB treatment decreased the cell viability in a dose-dependent manner (Figure 1C, 1D). To evaluate the cytoprotective properties of galangin following H_2_O_2_ and UVB exposure, we treated HS68 cell with different concentration of galangin (10, 20, 30 μM) following H_2_O_2_- (200 μM) and UVB-induced (40 mJ/cm²) damage. H_2_O_2_ or UVB treatment alone significantly decreased (40%) the viability of HS68 cells. However, galangin treatment prevented cell death in a concentration-dependent manner (Figure 1E, 1F). Higher concentration of galangin (30 μM) prominently alleviated H_2_O_2_- and UVB-induced cytotoxicity. Therefore, the dose of 30 μM galangin was used in subsequent *in vitro* studies to evaluate its protective effects in HS68 cells.

### Galangin attenuates H_2_O_2_-induced HS68 cell senescence rather than apoptosis

**Figure 2:**
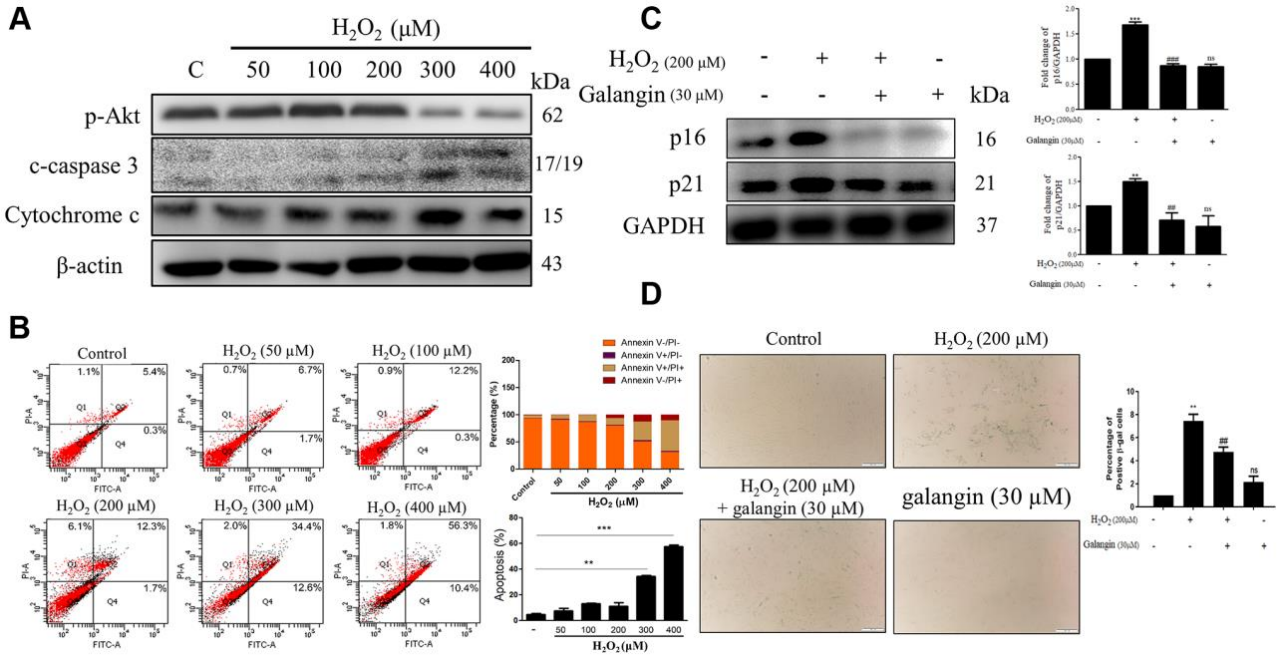
**Galangin attenuates H_2_O_2_-induced HS68 cell senescence rather than apoptosis**. (**A**) HS68 cells were exposed to different doses of H_2_O_2_ (50, 100, 200, 300, 400 μM) for 24 h. Apoptosis and survival-related markers were detected by western blot. (**B**) Annexin V & PI stained cells were analyzed by flow cytometry to identify the apoptotic cells under H_2_O_2_ exposure. (**C**) HS68 cells were exposed to H_2_O_2_ (200 μM) for 1 h and then treated with galangin (30 μM) for 23 h. Aging markers such as p16 and p21 were detected by western blot. GAPDH was used as a loading control. (**D**) Senescent cells were detected using a senescence-associated β-galactosidase (SA-β-gal) detecting kit. SA-β-gal-positive cells are shown in green color. (**C**) Values are shown as mean ± SE. Quantification of the results is shown (*n* = 3) ^**^P < 0.01, ^***^P < 0.001 vs. untreated control cells; ^##^*P* < 0.01, ^###^*P* < 0.001 vs. H_2_O_2_-treated cells.

To determine whether decrease in cell viability was due to cell death or growth inhibition, we checked the expression of several apoptosis and survival markers by western blotting in HS68 cells exposed to different doses of H_2_O_2_ (50, 100, 200, 300, 400 μM) for 24 h. We found significantly decreased expression of survival markers, such as p-Akt, and increased expression of apoptosis-related markers, such as cleaved-caspase 3 and cytochrome c, at concentrations >200 μM H_2_O_2_ (Figure 2A). Furthermore, we performed flow cytometry to assess apoptotic cells using Annexin V and PI staining and observed similar results. Taken together, 200 μM H_2_O_2_ did not cause HS68 cell apoptosis (Figure 2B). Next, we detected the expression of several senescence markers, such as p16, p21, and SA-β-gal, to determine the senescent cells. Our results showed that galangin treatment significantly decreased the H_2_O_2_-induced p16 and p21 protein levels as well as number of SA-β-gal-positive cells (Figure 2C, 2D).

### Galangin attenuates UVB/H_2_O_2_-induced intracellular/mitochondrial ROS production and MMP imbalance in HS68 cells

**Figure 3:**
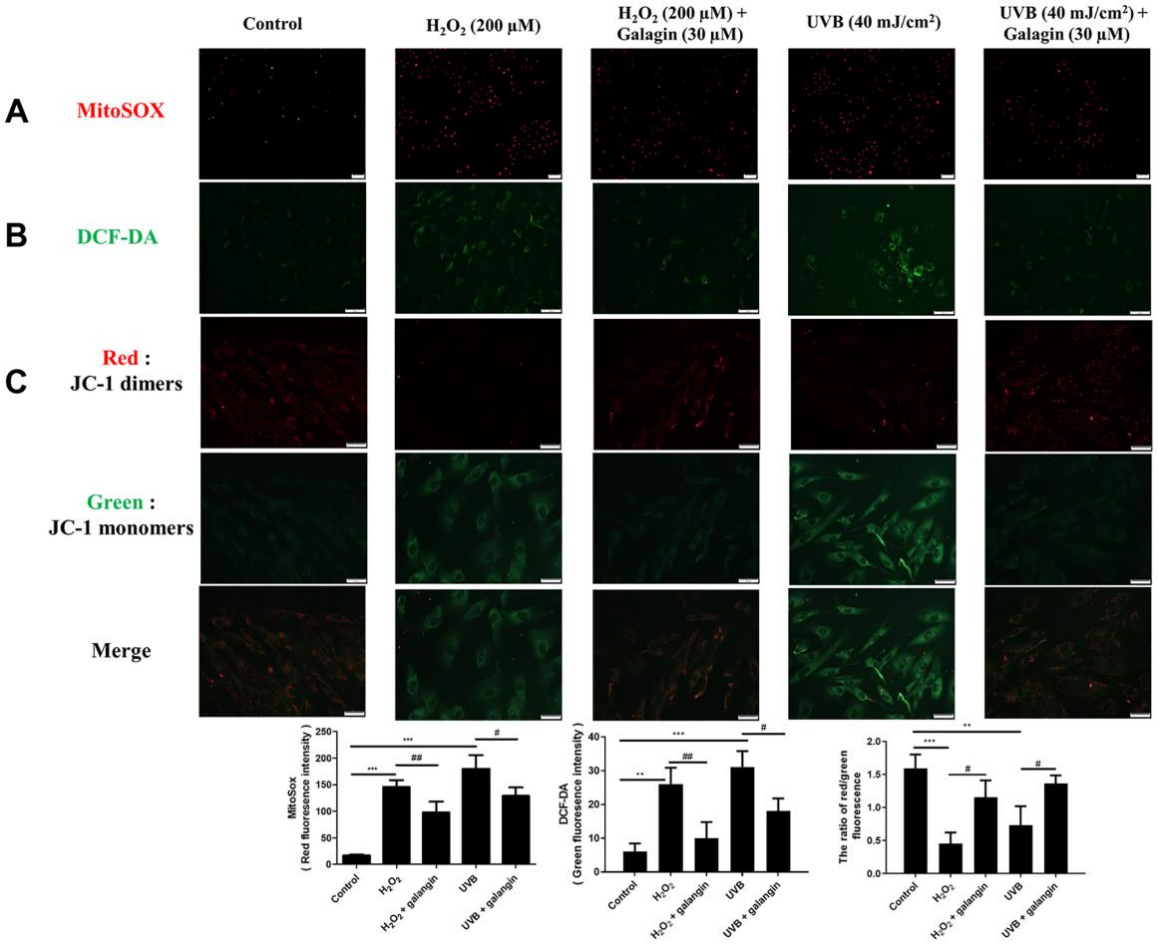
**Galangin attenuates UVB/H_2_O_2_-induced intracellular and mitochondrial ROS production as well as MMP imbalance in HS68 cells.** HS68 cells were exposed to H_2_O_2_ (200 μM) for 1 h and then co-treated with galangin (30 μM) for 23 h. HS68 cells were exposed to UVB radiation (40 J/cm^2^) and then co-treated with galangin (30 μM) for 24 h. (**A**, **B**) Intracellular and mitochondrial ROS levels were determined by MitoSOX™ red mitochondrial superoxide indicator (2 μM) and 2′,7′-dichlorofluorescin diacetate (DCF-DA) (5 μM). The mitochondria positive for ROS fluoresced red, while green fluorescence indicated intracellular ROS levels. (**C**) Mitochondrial membrane potential (MMP) was determined by JC-1 staining. Red fluorescence indicated JC-1 dimers (intact mitochondria) and green fluorescence indicated JC-1 monomers (impaired mitochondria). The images were taken using a florescence microscope. Values are shown as mean ± SE. Quantification of the results is shown (*n* = 3) ^**^P < 0.01, ^***^P < 0.001 vs. untreated control cells; ^#^*P* < 0.05, vs. H_2_O_2_ or UVB-treated cells.

Oxidative stress plays a key role in various diseases, and can be induced by endogenous or exogenous factors. Here, we used H_2_O_2_ as an endogenous and UVB as an exogenous factor to induce oxidative damage and then investigated the ROS eliminating activities of galangin. To identify the sources of ROS, MitoSOX and DCFH-DA were used to determine intracellular and mitochondrial ROS. The staining results showed that H_2_O_2_ and UVB treatment induced ROS accumulation while galangin treatment reduced ROS generation (Figure 3A, 3B). In addition, as imbalanced MMP can initiate cell death, we used JC-1 to determine changes in MMP. Normal mitochondria fluoresced red (JC-1 dimers) while impaired mitochondria fluoresced green (JC-1 monomers). UVB and H_2_O_2_ exposure induced high levels of green fluorescence. Notably, galangin treatment shifted the green fluorescence to red, indicating revival of mitochondrial functions (Figure 3C).

### Galangin attenuates H_2_O_2_-induced TGFβ/Smad collagen synthesis pathway impairment in HS68 cells

**Figure 4:**
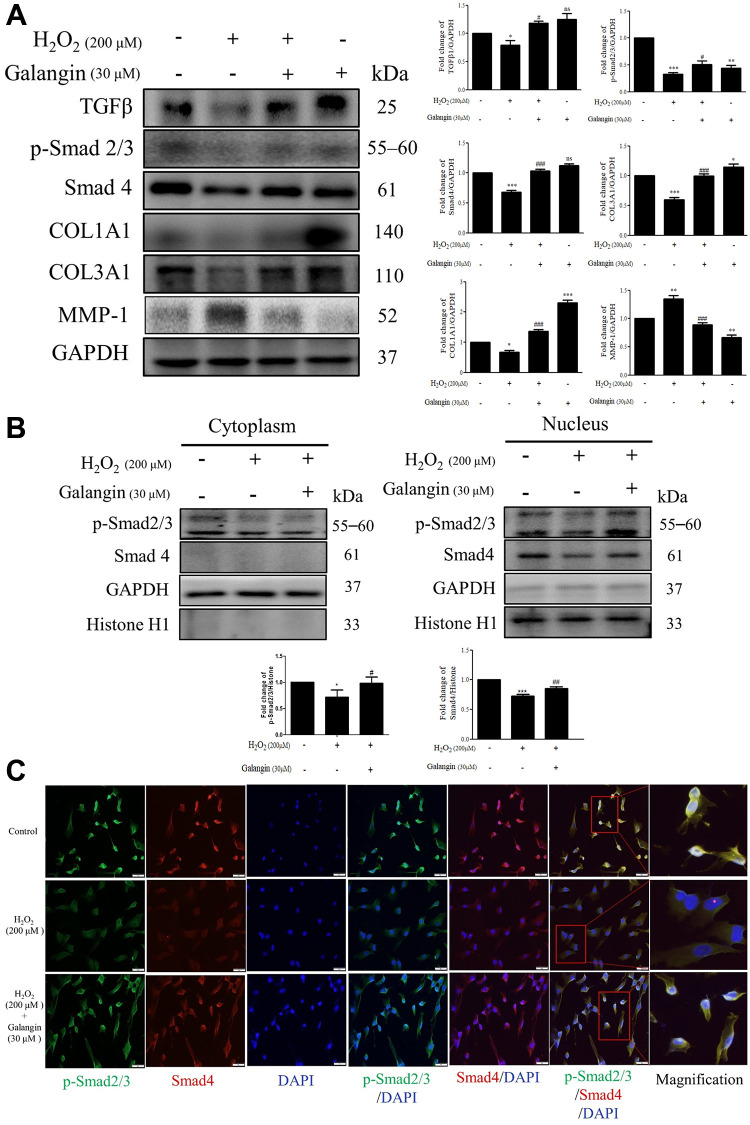
**Galangin attenuates H_2_O_2_-induced TGFβ/Smad collagen synthesis pathway impairment in HS68 cells.** (**A**) HS68 cells were exposed to H_2_O_2_ (200 μM) for 1 h and then treated with galangin (30 μM) for 23 h. Protein expression of collagen synthesis-related pathway components (TGFβ, p-smad2/3, Smad4, COL1A1, COL3A1) and collagen degradation-related protein (MMP-1) were detected by western blot. (**B**) The protein expression of p-smad2/3, Smad4 in the nuclear and cytosolic fractions was detected by western blotting. GAPDH was used as a loading control. Values are shown as mean ± SE. Quantification of the results is shown (*n* = 3) ^*^P < 0.05, ^**^P < 0.01, ^***^P < 0.001 vs. untreated control cells; ^#^*P* < 0.05, ^##^*P* < 0.01, ^###^*P* < 0.001 vs. H_2_O_2_-treated cells. (**C**) Anti-p-Smad2/3, Smad4 antibody, and FITC/PE-conjugated secondary antibody were used to detect p-Smad2/3 (green), Smad4 (red) expression. DAPI indicated the nucleus location (blue). The images were captured using a florescence microscope (200×).

TGFβ/Smad signaling is conducive to collagen formation in human skin dermal fibroblasts [25–27]. Therefore, we investigated the role of galangin in collagen synthesis via TGFβ/Smad pathway in HS68 cells exposed to H_2_O_2_. Western blot results indicated that galangin significantly enhanced TGFβ/Smad signaling as well as collagen synthesis in H_2_O_2_-exposed HS68 cells (Figure 4A). Moreover, disorganization of collagen type I and III is one of the typical characteristics of aged skin fibroblasts [28]. Since the MMP family has been implicated in collagen catabolism [29], we next confirmed the influence of galangin on MMP-1 activation. We found that galangin prevented collagen degradation in HS68 cells by suppressing H_2_O_2_-enhanced MMP-1 level (Figure 4A). Furthermore, we identified the subcellular molecular mechanism underlying this protective action by comparing HS68 cells treated with H_2_O_2_ alone or co-treated with galangin using immunoblotting and immunofluorescence staining. Galangin treatment enhanced nuclear accumulation of p-smad2/3 and Smad4 that was disrupted by exposure to H_2_O_2_ in HS68 cells (Figure 4B, 4C). These findings indicated that galangin may ameliorate H_2_O_2_-induced collagen fragmentation by activating TGFβ/Smad pathway in HS68 cells.

### Galangin treatment reduces the H_2_O_2_-induced upregulated expression of hsa-miR-4535, predicted to target Smad4

**Figure 5:**
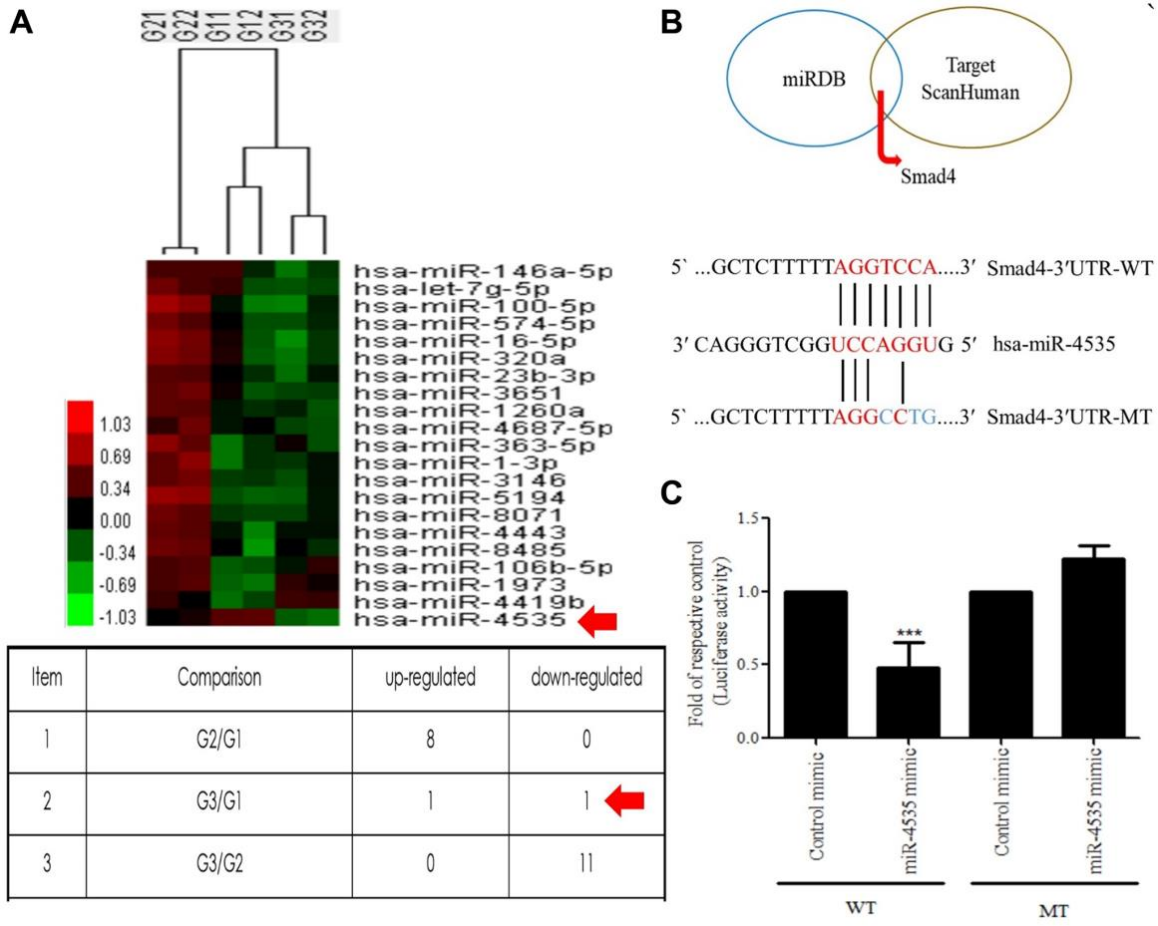
**Hsa-miR-4535 targets Smad4.** (**A**) MicroRNA expression in HS68 cells treated with galangin was assessed using microarrays. (**B**) Venn diagram depicting potential mRNA candidates that may be regulated by hsa-miR-4535 based on miRDB and TargetScanHuman databases. Sequence analysis of putative miRNA-binding sites in Smad4-3′-UTR mRNA for hsa-miR-4535. Matches are indicated by straight lines. (**C**) HS68 cells were co-transfected with Smad4-3′-UTR-WT (wild-type; 0.5 μg/mL) plus hsa-miR-4535 mimic (20 nM) or Smad4-3′-UTR-MT (mutant; 0.5 μg/mL) plus hsa-miR-4535 mimic (20 nM) for 24 h. Luciferase activity was determined and normalized to that of Renilla. Quantification of the results is shown (*n* = 3); ^***^P < 0.001, versus the Smad4-3′-UTR-MT plus hsa-miR-4535 mimic group. G1 = control, G2 = galangin (10 μM), G3 = galangin (30 μM).

**Figure 6:**
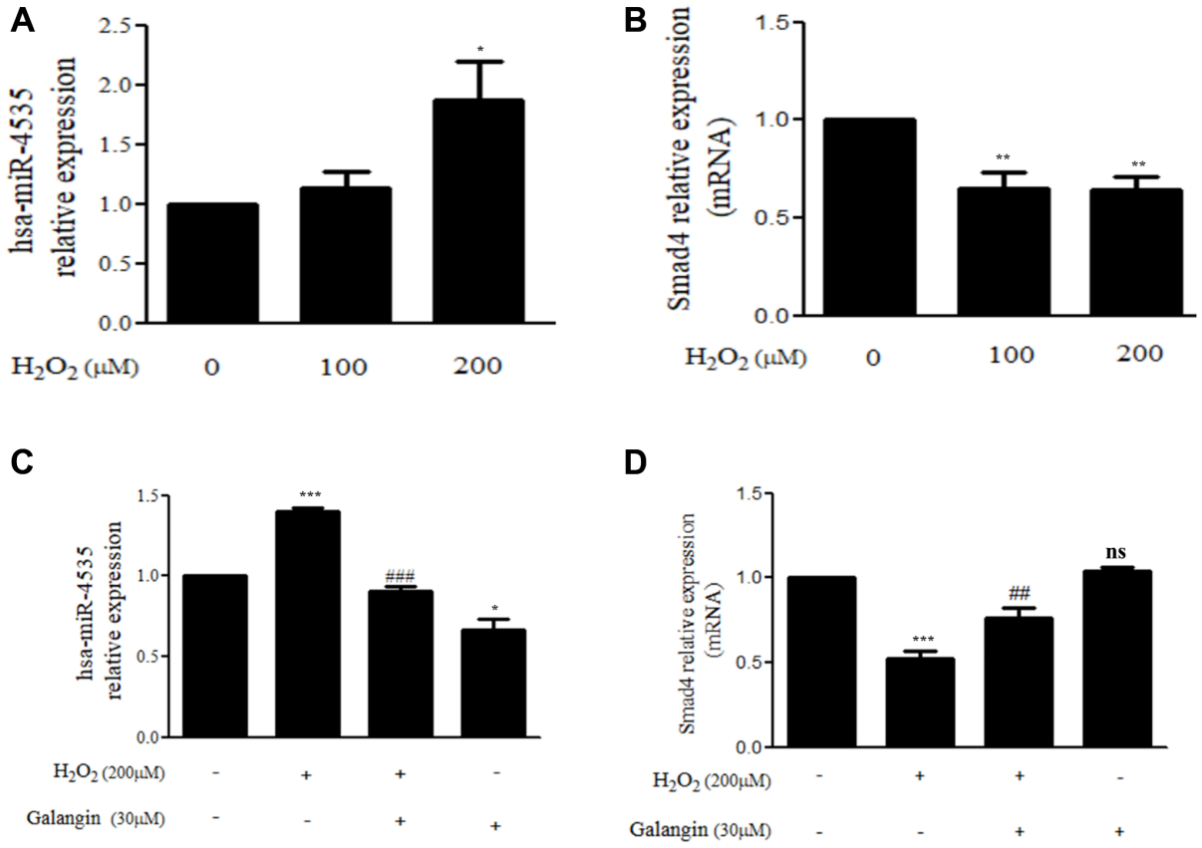
**Galangin up-regulates Smad4 expression by decreasing hsa-miR-4535 level.** (**A**, **B**) HS68 cells were treated with different concentrations of H_2_O_2_ (0, 100, 200 μM) for 24 h. (**C**, **D**) HS68 cells were exposed to H_2_O_2_ (200 μM) for 1 h and then treated with galangin (30 μM) for 23 h. The expression of hsa-miR-4535 and Smad4 were detected by qRT-PCR. Values are shown as mean ± SE. Quantification of the results is shown (*n* = 3) ^*^P < 0.05, ^**^P < 0.01, ^***^P < 0.001 vs untreated control cells; ^##^*P* < 0.01, ^###^*P* < 0.001 vs H_2_O_2_-treated cells.

The miRNA array analysis of galangin-treated HS68 cells revealed that hsa-miR-4535 expression was reduced in galangin-treated group as compared to that in the control group (Figure 5A). Further, using miRNA databases, such as miRDB and TargetScanHuman, we predicted a putative conserved target site for hsa-miR-4535 in Smad4-3′-UTR (Figure 5B). After confirming the target site, Smad4-3′-UTR-WT and Smad4-3′-UTR-MT reporter constructs were transfected into HS68 cells to verify the direct binding of hsa-miR-4535 to Smad4-3′-UTR. We observed 50% reduction in luciferase activities between wild type and mutant Smad4-3′-UTR groups followed hsa-miR-4535 mimic transfections (Figure 5C). We then assessed the effects of H_2_O_2_ (100, 200 μM) on the regulation of hsa-miR-4535 and Smad 4 in HS68 cells, and found that H_2_O_2_ dose-dependently enhanced hsa-miR-4535 and suppressed Smad4 RNA expression (Figure 6A, 6B). In addition, this trend in hsa-miR-4535 and Smad4 expression was reversed following galangin treatment, indicating that regulation of Smad signaling may be involved in the galangin-mediated alleviation of H_2_O_2_-induced HS68 cell damage (Figure 6C, 6D). Taken together, these results suggested that galangin attenuates H_2_O_2_-induced TGFβ/Smad pathway impairment by downregulating hsa-miR-4535 expression in human skin dermal fibroblasts.

### Modulation of Smad4 by hsa-miR-4535 is involved in collagen synthesis in H_2_O_2_-exposed human skin dermal fibroblasts

**Figure 7:**
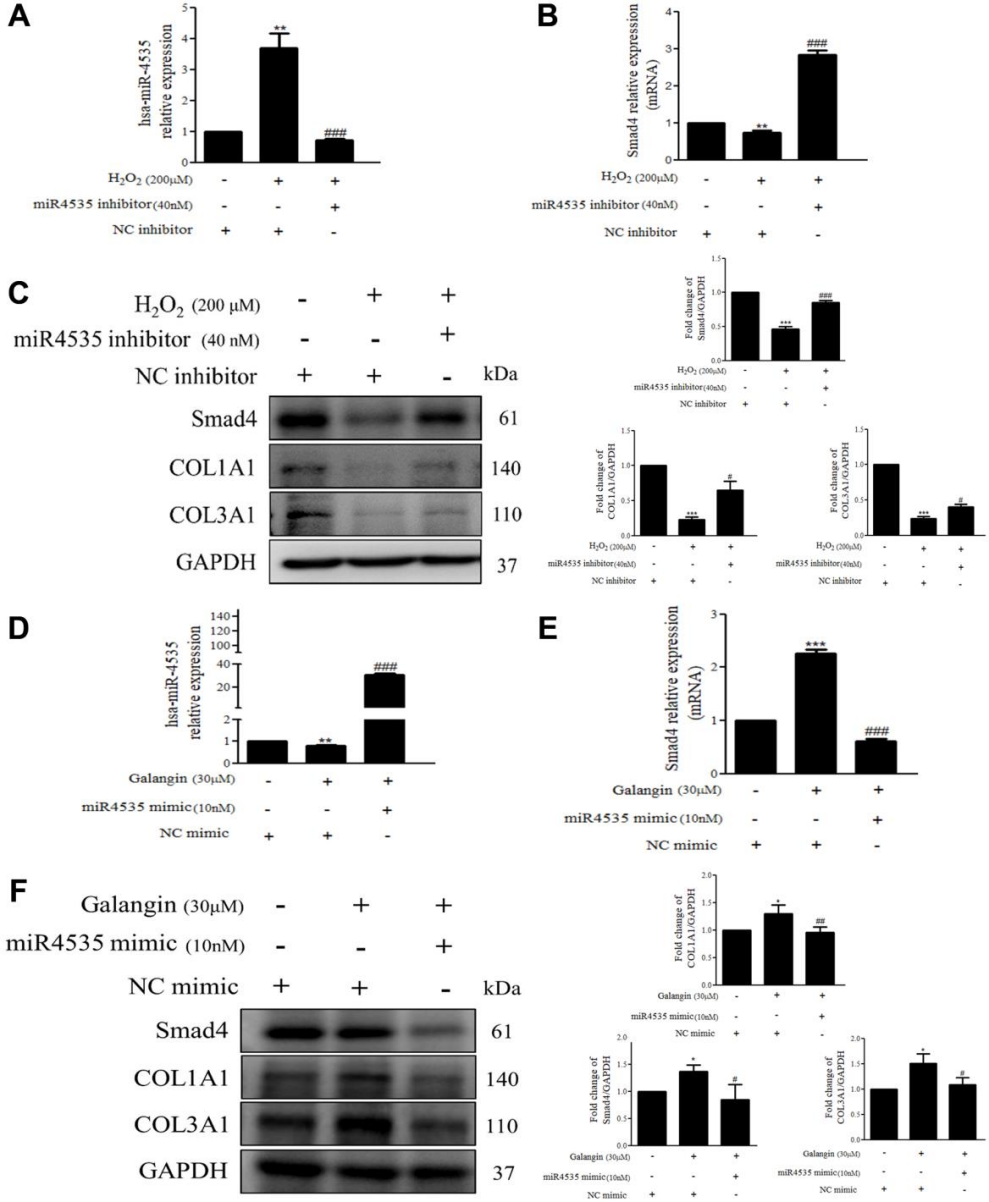
**Overexpression or inhibition of hsa-miR-4535 regulates collagen synthesis in HS68 cells.** (**A**–**C**) HS68 cells were transfected with hsa-miR-4535 inhibitor (40 nM) or inhibitor NC (40 nM) for 1 h and then cotreated with H2O2 (200 μM) for 23 h. (**D**–**F**) HS68 cells were transfected with hsa-miR-4535 mimic (10 nM) or mimic NC (10 nM) for 1 h and then co-treated with galangin (30 μM) for 23 h. hsa-miR-4535 levels were detected by qPCR to ensure successful transfection. Protein levels of Smad4, COL1A1, and COL3A1 were analyzed by western blotting. GAPDH was used as a loading control. Values are shown as mean ± SE. Quantification of the results is shown (*n* = 3) ^*^P < 0.05, ^**^P < 0.01, ^***^P < 0.001 vs. untreated control cells; ^#^*P* < 0.05, ^##^*P* < 0.01, ^###^*P* < 0.001 vs. H_2_O_2_ or galangin-treated cells.

**Figure 8:**
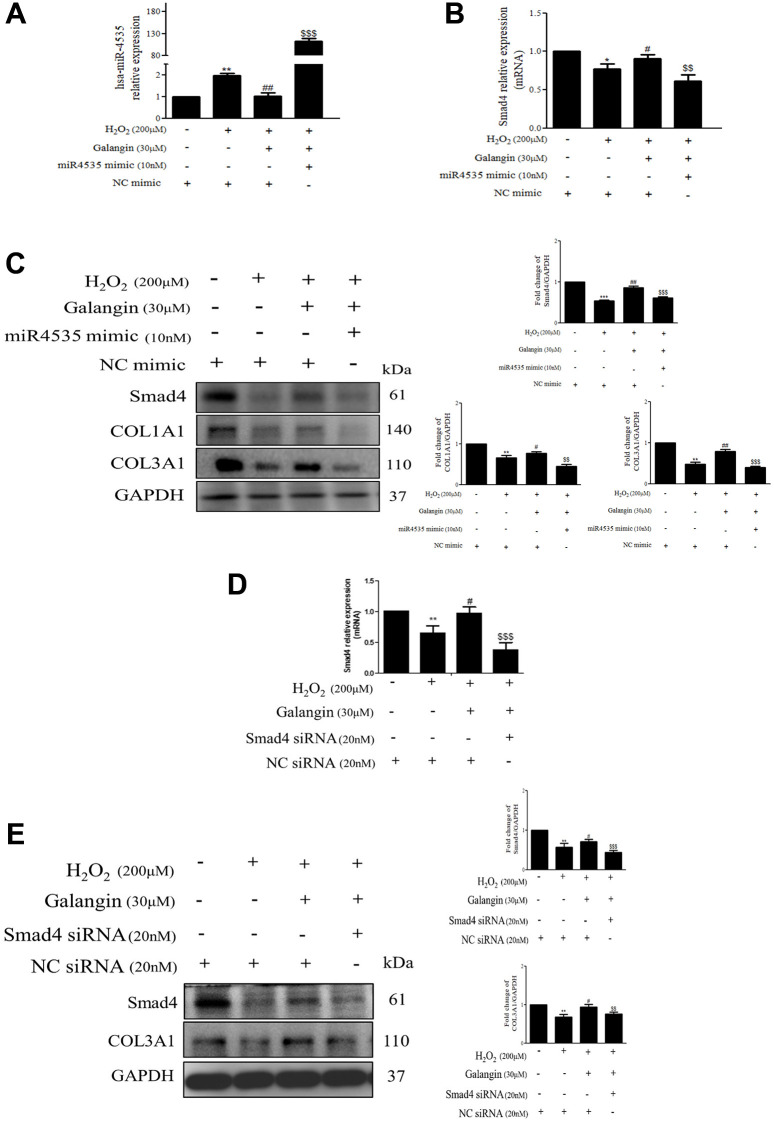
**Inhibition of Smad4 by siRNA or hsa-miR-4535 mimic reverses the galangin-mediated enhancement of collagen synthesis in dermal fibroblasts.** (**A**–**C**) HS68 cells were transfected with hsa-miR-4535 mimic (10 nM) or mimic NC (10 nM) for 1 h followed by cotreatment with galangin (30 μM) and H_2_O_2_ (200 μM) for 23 h. Hsa-miR-4535 (**A**) and Smad4 (**B**) levels were detected by qPCR to ensure successful transfection. (**D**–**E**) HS68 cells were transfected with siRNA Smad4 (20 nM) or siRNA NC (20 nM) for 1 h followed by cotreatment with galangin (30 μM) and H_2_O_2_ (200 μM) for 23 h. Smad4 levels were detected by qPCR to ensure successful transfection (**D**). Protein levels of Smad4, COL1A1 and COL3A1 were analyzed by western blotting (**C**, **E**). GAPDH was used as a loading control. Values shown are means ± SE. Quantification of the results is shown (*n* = 3) ^*^P < 0.05, ^**^P < 0.01, ^***^P < 0.001 vs. untreated control cells; ^#^*P* < 0.05, ^##^*P* < 0.01, vs. H_2_O_2_-treated cells; ^$$^*P* < 0.01, ^$$$^*P* < 0.001 vs. H_2_O_2_ plus galangin-treated cells.

To further determine the mechanisms by which hsa-miR-4535 exerts its effect on the expression of Smad4 and its downstream signaling in HS68 cells, we transfected HS68 cells with hsa-miR-4535 mimics or inhibitors and measured Smad4 and its downstream collagen expression. The results demonstrated that inhibition of hsa-miR-4535 by hsa-miR-4535 inhibitor significantly reversed the downregulation of Smad4 mRNA and protein expression in H_2_O_2_-treated HS68 cells (Figure 7A–7C). Moreover, suppression of hsa-miR-4535 partially restored H_2_O_2_-induced COL1A1 and COL3A1 impairment in H_2_O_2_-treated HS68 cells (Figure 7C). Conversely, enhanced hsa-miR-4535 expression as a result of hsa-miR-4535 mimic transfection blocked the galangin-mediated upregulation of Smad4 in HS68 cells exposed to H_2_O_2_ (Figure 7D–7F). Overexpression of hsa-miR-4535 partially reduced galangin-induced COL1A1 and COL3A1 levels (Figure 7F). The overexpression of hsa-miR-4535 (Figure 8A–8C) or inhibition of Smad4 by siRNA (Figure 8D, 8E) resulted in reduced collagen synthesis under galangin treatment following H_2_O_2_ exposure. Surprisingly, we found that both direct Smad4 silencing and treatments with hsa-miR-4535 mimic reversed galangin-induced collagen synthesis in H_2_O_2_-treated HS68 cells. Taken together, we concluded that galangin regulated collagen synthesis, potentially via decreasing hsa-miR-4535 level to enhance Smad4 signaling in HS68 cells exposed to H_2_O_2_.

### Galangin enhances TGFβ/Smad collagen synthesis pathway and attenuates dermal senescence as well as inhibition of hsa-miR-4535 expression in UVB-exposed HS68 cells

**Figure 9:**
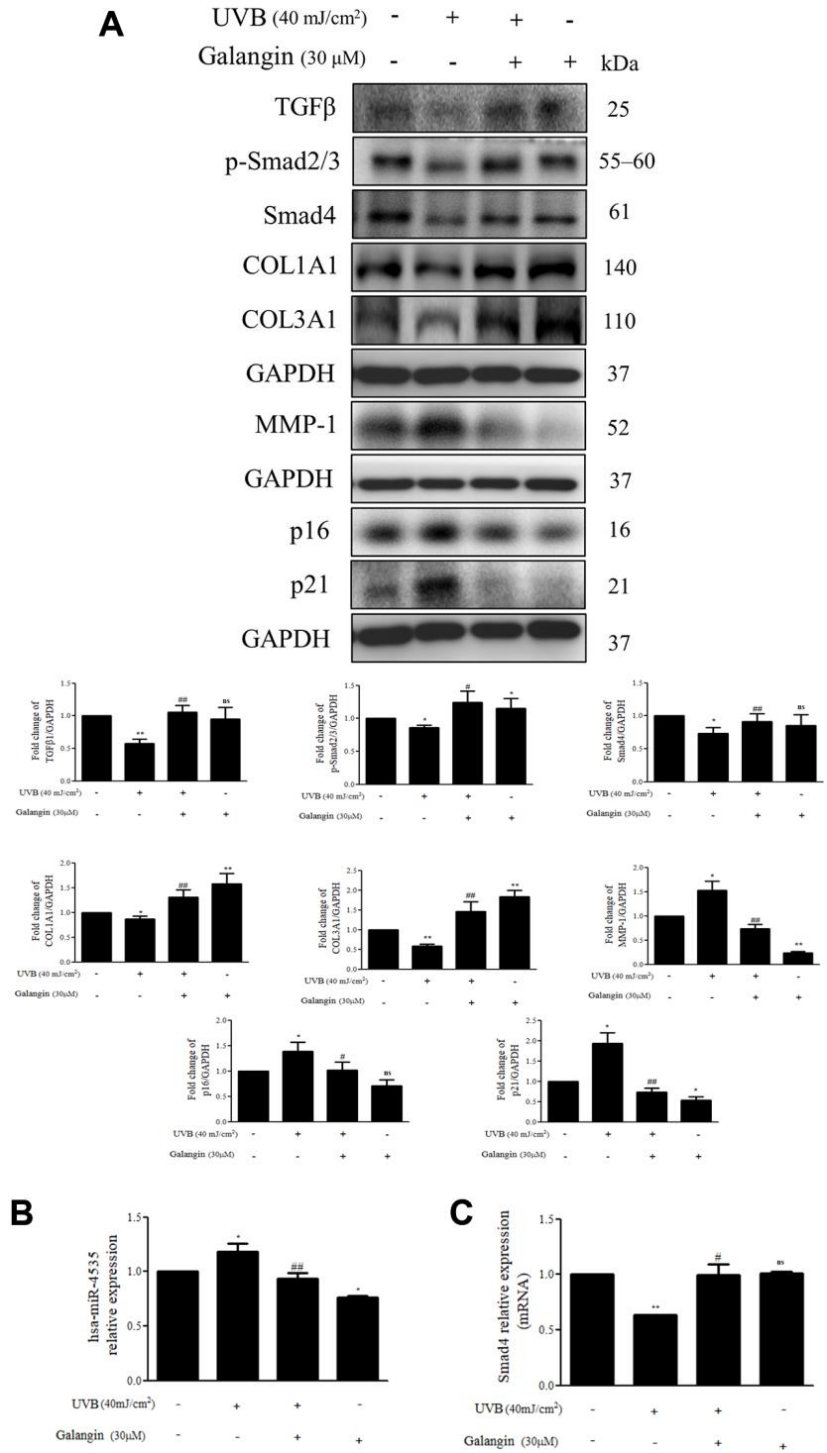
**Galangin enhances TGFβ/Smad collagen synthesis pathway and attenuates dermal senescence as well as inhibition of hsa-miR-4535 expression in UVB-exposed HS68 cells.** HS68 cells were exposed to UVB (40 mJ/cm^2^) and then treated with galangin (30 μM) for 23 h. (**A**) Protein expression of collagen synthesis-related pathway components (TGFβ, p-smad2/3, Smad4, COL1A1, COL3A1), collagen degradation-related protein (MMP-1), and senescence-associated markers (p16 and p21) was detected by western blot. GAPDH was used as a loading control. (**B** and **C**) RNA expression of hsa-miR-4535 and Smad4 was detected by qRT-PCR. Values are shown as mean ± SE. Quantification of the results is shown (*n* = 3) ^*^P < 0.05, ^**^P < 0.01, vs untreated control cells; ^#^*P* < 0.05, ^##^*P* < 0.01 vs UVB-exposed cells.

As UV-light plays an important role in various skin dermis disorders, including inflammation, immunosuppression, cancer, and premature aging [30–32], we examined the effect of galangin on collagen synthesis-related signaling in HS68 cells exposed to UVB radiation. The protein levels of TGFβ/Smad pathway components and the downstream COL1A1 and COL3A1 were decreased in UVB-treated HS68 cells but were effectively upregulated following galangin treatment (Figure 9A). The western blotting results confirmed that galangin suppressed UVB-enhanced MMP-1 expression in HS68 cells (Figure 9A). Moreover, we clarified the anti-aging effect of galangin in HS68 cells subjected to UVB exposure by demonstrating that galangin reduced UVB-induced elevation of p16 and p21 levels (Figure 9A). To assess whether galangin could attenuate UVB-induced collagen breakdown through inhibition of hsa-miR-4535 targeting Smad4, we investigated hsa-miR-4535 and Smad4 levels in UVB and galangin co-treated cells using qPCR. We found that hsa-miR-4535 expression was significantly upregulated in UVB-exposed cells but decreased following galangin treatment. Conversely, Smad4 expression was suppressed by UVB exposure and this suppression was markedly reversed by galangin treatment (Figure 9B, 9C). Collectively, our findings suggested that galangin exerts similar effects as H_2_O_2_ to restore UVB-induced decrease in collagen formation by repressing hsa-miR-4535 levels so as to promote Smad4 signaling in HS68 cells.

### Topical application of galangin attenuates UVB-induced skin hyperplasia and collagen degradation as well as enhances TGFβ/Smad signaling in the dorsal skin of C57BL6/J nude mice

**Figure 10:**
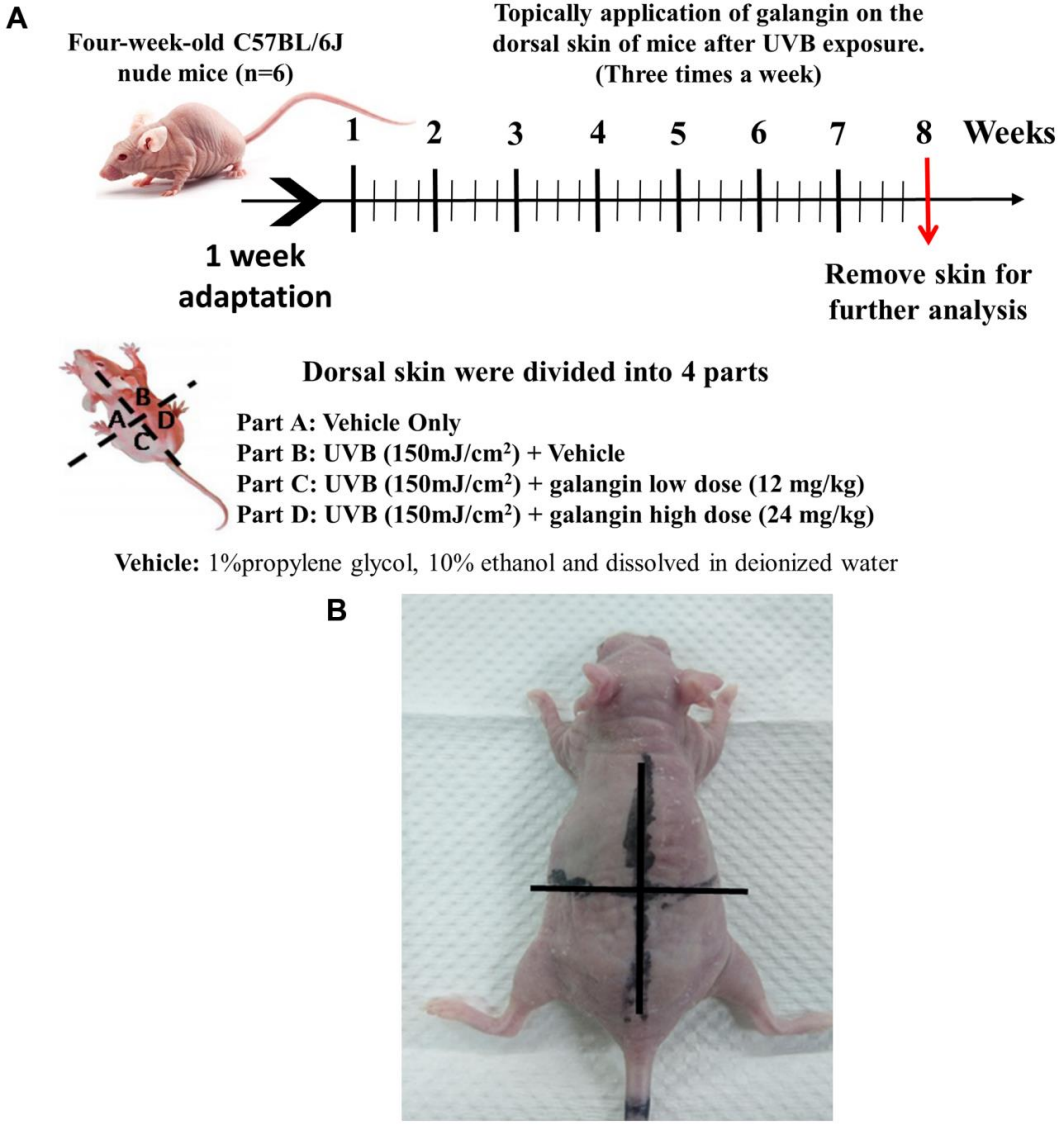
**Effect of galangin on UVB-induced C57BL6/J nude mouse skin photodamage.** (**A**) The schematic procedure of animal experiments. Dorsal skin of four-week-old C57BL6/J nude mice (*n* = 6) was exposed to UVB radiation and treated with galangin once every two days for 8 weeks (**B**) Photograph depicting wrinkle formation on the dorsal skin of nude mice due to UVB exposure, followed by topical application of galangin.

**Figure 11:**
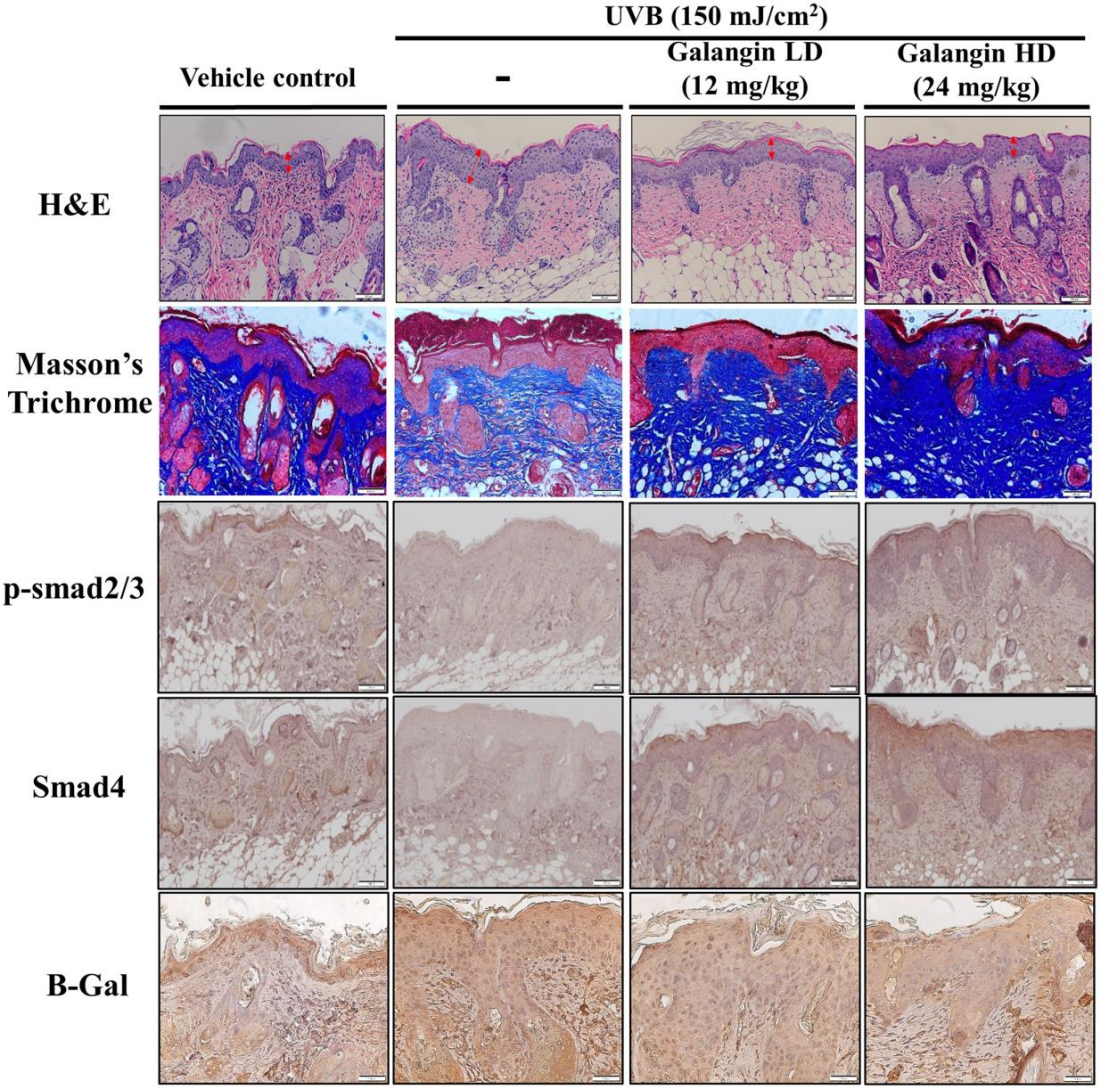
**Topical galangin application attenuates UVB-induced skin hyperplasia and collagen degradation in the dorsal skin of C57BL6/J nude mice.** Skin tissues were serially sectioned and stained with H&E, Masson’s trichrome, and immunohistochemically for p-Smad2/3 Smad4 and β-gal. H&E staining showed epidermal thickness. Double-headed red arrows indicate the epidermal thickening. Masson’s trichrome staining showed the collagen fiber content in dermal layers. Collagen fibers appear blue. LD, low dose; HD, high dose.

**Figure 12:**
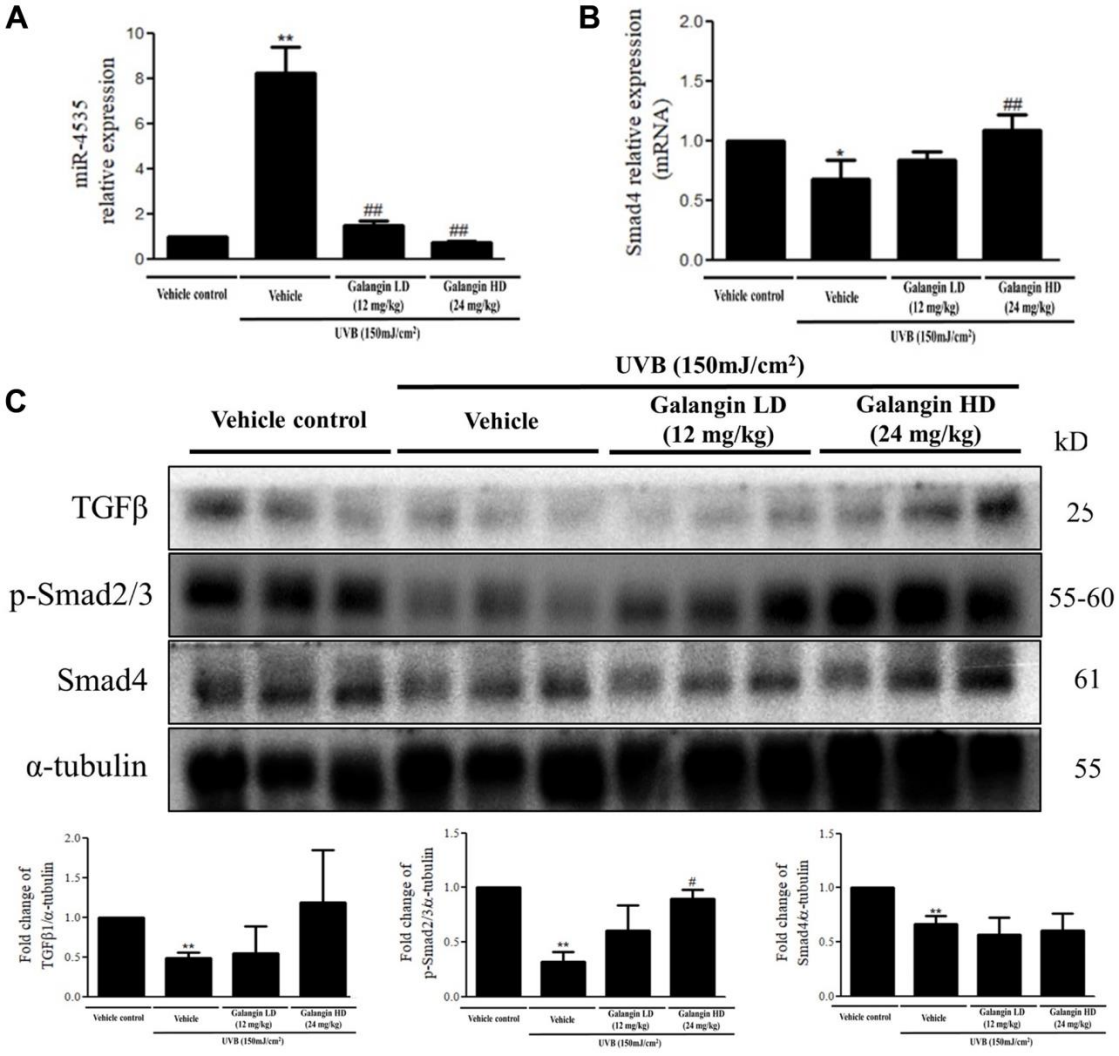
**Effects of galangin on the TGFβ/Smad signaling and hsa-miR-4535 expression in UVB-irradiated mice.** (**A** and **B**) mRNA levels of hsa-miR-4535 and Smad4 from dorsal skin of UVB-exposed mice were detected by qRT-PCR. (**C**) The protein levels of TGFβ, p-smad2/3, and Smad4 in dorsal skin tissues were detected by western blotting. α-tubulin was used as a loading control. Data are presented as mean ± SD. Quantification of the results are shown; ^*^P < 0.05, ^**^P < 0.01 versus vehicle control group; ^#^*P* < 0.05, ^##^*P* < 0.01 versus UVB plus vehicle group.

**Figure 13:**
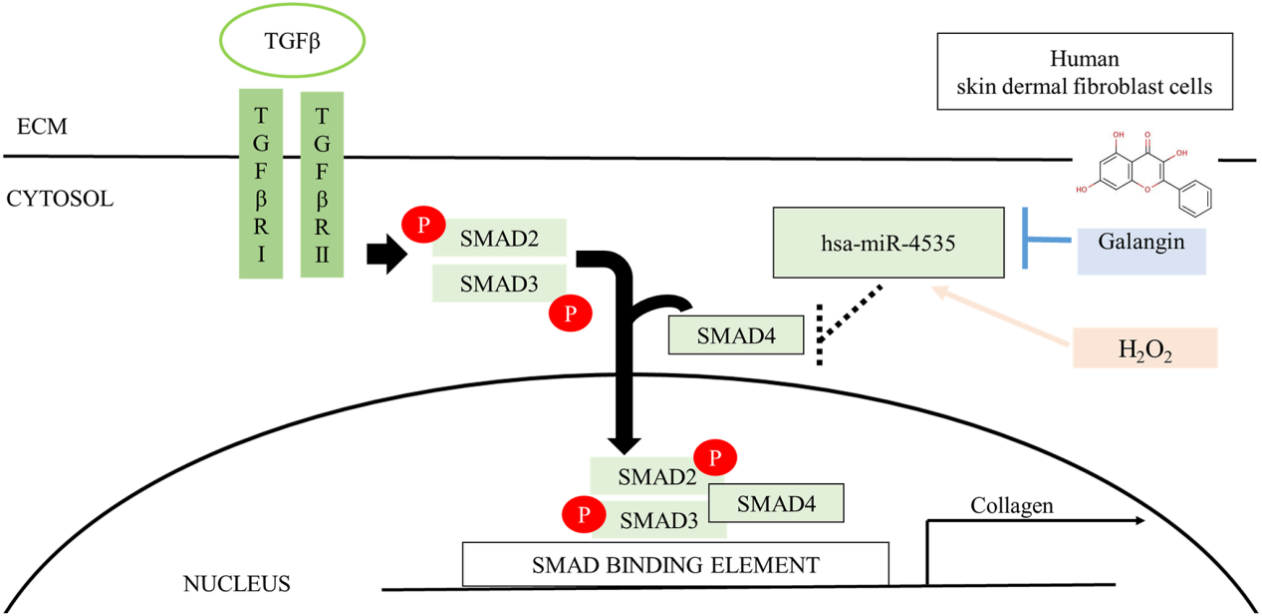
**Schematic diagram for the mechanism of galangin action.** Galangin enhances TGFβ/Smad collagen synthesis pathways by reducing H_2_O_2_-induced hsa-microRNA-4535 to improve photoaging in human skin dermal fibroblast. Abbreviations: ECM: extracellular matrix; TGFβ: transforming grown factor beta; TβRI: TGFβ receptor type I; TβRII: TGFβ receptor type II.

To elucidate the photo-protective effect of galangin on UVB-induced skin injury *in vivo*, we evaluated the level of microRNA-4535, Smad4, and skin tissue alterations. Figure 10A shows the schematic for experimental design and time-line for topical application of galangin following UVB irradiation on the dorsal skin of C57BL6/J nude mice. The formation of wrinkles was observed on the dorsal skin sections of C57BL/6J mice in the UVB-exposed group. Topical application of galangin significantly reduced wrinkle formation, indicating the galangin-mediated increase in collagen content to maintain skin integrity (Figure 10B). Moreover, histological analysis of the dermal layer showed that collagen content and density in the skin were significantly lower in UVB-irradiated group compared to that in the vehicle control group. However, topical application of galangin (12 mg/kg and 24 mg/kg) promoted increased the collagen content and density in a dose-dependent manner compared with that in mice subjected to UVB-irradiation (Figure 11). The skin morphology was evaluated by H&E staining. UVB exposure increased epidermal thickness compared with vehicle control group. However, topical application of galangin significantly alleviated UVB-induced epidermal hyperplasia (Figure 11). In addition, the immunohistochemical staining of β-gal indicated galangin application reduced the high β-gal expression in UVB-irradiated skin (Figure 11). To determine whether galangin can ameliorate UVB-induced collagen fiber loss via hsa-miR-4535 downregulation to enhance Smad4 *in vivo*, its RNA expression was assessed in the excised dorsal skin sample by qRT-PCR. hsa-miR-4535 expression was elevated in UVB-irradiated skin but significantly suppressed in the galangin-treated skin at both low and high doses. Furthermore, the low levels of Smad4 RNA expression in the UVB-irradiated skin were restored by galangin treatment (Figure 12A, 12B). In addition, galangin application rescued TGFβ, p-smad2/3, and Smad4 protein expression in UVB-impaired skin tissue (Figure 12C). Our findings indicated that galangin could potentially protect the skin from UVB-induced damages by inhibiting hsa-miR-4535 to enhance Smad4 expression for activation of P- smad2/3 to finally promote collagen synthesis (Figure 13).

## DISCUSSION

In this study, we identified that the protective role of galangin is mediated through attenuation of hsa-miR-4535 that targets Smad4, thus regulating Smad2/3/4 signaling as well as collagen synthesis in the skin exposed to H_2_O_2_/UVB-induced dermal damage *in vitro* and *in vivo*. Our findings can be summarized as six main points: (1) galangin attenuated H_2_O_2_-induced HS68 cell senescence but not apoptosis at 30 μM dose; (2) H_2_O_2_/UVB-induced intracellular and mitochondrial ROS formation and MMP imbalance were reversed following galangin treatment; (3) galangin significantly enhanced TGFβ/Smad signaling as well as Samd2/3/4 complex activation in H_2_O_2_-exposed cells; (4) upregulation of hsa-miR-4535 by H_2_O_2_/UVB was suppressed following galangin treatment; (5) hsa-miR-4535 is involved in regulating TGFβ/Smad signaling by targeting Smad4 to impaired collagen synthesis in H_2_O_2_/UVB-exposed cells; (6) topical application of galangin on the dorsal skin of nude mice significantly reduced UVB-induced skin photoaging, wrinkle formation, skin hyperplasia, and the impairment of TGFβ/Smad signaling pathways.

The human skin is comprised of two layers: epidermis and dermis. The skin dermis contains connective tissue, hair follicles, and sweat glands. Human skin dermal fibroblasts are embedded in the dermis [33]. Various studies have indicated that intrinsic and extrinsic aging both result in skin dermal fibroblast senescence due to accumulation of ROS [16, 34, 35]. Thus, aged human dermal fibroblasts irreversibly arrest cell cycle and lose their proliferative capacities [36]. H_2_O_2_ or UVB irradiation are well-characterized sources for accelerating ROS production in human dermal fibroblasts [37, 38]. Here, we found that galangin (10–50 μM) treatment did not cause cytotoxicity but rather substantially attenuated H_2_O_2_/UVB -induced cell death in human dermal fibroblasts (Figure 1E, 1F). Furthermore, galangin treatment suppressed H_2_O_2_/UVB-induced upregulation of p16, p21 and the number of SA-β-gal-positive cells (Figure 2D). Thus, the demo-protective effects of galangin against H_2_O_2_ or UVB damage might be linked to the suppression of different sources of ROS or integrity of the mitochondria.

Galangin reportedly exhibited anti-apoptotic and antioxidant properties in various cells such as keratinocytes and cardiomyocytes [13, 39, 40]. Oral administration of galangin effectively suppressed streptozotocin-induced liver injury in diabetic rats by reducing mitochondrial oxidative damage and enhancing Nrf2 antioxidant signaling [13, 41]. Other studies have showed that galangin ameliorated cyclophosphamide-induced hepatotoxicity and cisplatin-induced nephrotoxicity by attenuating oxidative stress and inflammation [42, 43]. Exposure of dermal fibroblasts to UVB or H_2_O_2_ results in accumulation of ROS in mitochondria, leading to mitochondrion fragmentation and cellular aging [44]. A previous study showed that fragmented mitochondria impair collagen production [45]. Our study indicated that galangin possesses radical scavenging ability to prevent ROS-induced mitochondrion membrane potential imbalanced and ROS accumulation in UVB- and H_2_O_2_-induced cells. (Figure 3A–3C). Recent studies have reported that dermal fibroblasts are responsible for organizing ECM-rich environment, predominantly consisting of collagen types I and III [46]. The homeostasis of collagen types I and III is essentially responsible for maintaining structural and mechanical support for the skin [47]. Emerging evidences have suggested that abundant ROS are generated during aging, resulting in progressive loss of collagen bundles in the dermal layers and thus, contributing to wrinkle formation due to impaired TGFβ/Smad pathway and elevated MMP expression [48, 49]. Galangin-mediated activation of TGFβ/Smad signaling produces collagen that maintains skin integrity. However, it has different effects in cancers cells; in liver cancer, galangin-treatment inhibited HepG2 cell proliferation by activating TGFβ/Smad signaling [50]. In contrast, galangin inhibited TGFβ/Smad signaling as well as inactivated Smad3 resulting in growth inhibition in prostate and pancreatic cancer cells [51]. However, the role of galangin in TGFβ/Smad signaling in skin dermal fibroblasts remained unknown. Here, we observed that galangin upregulated COL1A1 and COL3A1 expression that was downregulated in H_2_O_2_/UVB-treated cells through activation of TGFβ/Smad signaling (Figure 4A–4C, 8E, 9A). Moreover, induction of MMP-1 by H_2_O_2_/UVB was reversed following galangin treatment (Figure 4A, 9A). Our findings indicate that galangin protects dermal fibroblasts from H_2_O_2_/UVB-induced damage by activating TGFβ/Smad signaling and inducing collagen production.

Skin dermis collagen development is a complicated physiological process, and microRNA-mediated regulation has been shown to contribute to the remodeling and organization of the ECM [23, 52, 53]. Collagen, elastin, and hyaluronic acid (HA) are the three main proteins that maintain skin integrity. Accumulating evidence suggests that several microRNAs are involved in dermal aging through ECM regulation. miR-181a was upregulated in senescent human dermal fibroblasts and enabled collagen XVI to modulate ECM components [54]. Interestingly, high expression of miR-23a-3p could induce dermal senescence by directly suppressing hyaluronan synthase 2, one of the transmembrane HA synthase isoenzymes that synthesizes HA [21]. Recently, various natural compounds have been reported to attenuate skin dermal aging via microRNA regulation [55–57]. Furthermore, galangin was reportedly involved in microRNA regulation in cholangiocarcinoma and hepatocellular carcinoma and caused suppression of cell growth and metastasis [58, 59]. So far, no study has identified the molecular mechanism, such as microRNA regulation, in galangin-treated human skin dermal fibroblasts cells. Here, we analyzed the microRNA array profiles to identify several microRNAs that differed between control and 30 μM galangin-treated groups (Figure 5A). Using miRDB and TargetScanHuman databases, we predicted Smad4 as the hsa-miR-4535 mRNA target (Figure 5B). hsa-miR-4535 expression was downregulated following galangin treatment in H_2_O_2_/UVB-induced cells (Figure 6C, 9B). In contrast, expression of its target, mRNA-Smad4, was significantly inhibited in H_2_O_2_/UVB-exposed cells and increased following galangin treatment (Figure 6D, 9C). A variety of studies indicated that ROS acts as an intermediate in stimulating stress-induced transcription factors, such as p53, NF-κB and HIF [60]. Therefore, we inferred that certain miRNAs could be modulated through the regulation of these transcription factors. By using transcription factor binding site database (PROMO), we identified that there is one putative p53 binding element at the hsa-miR-4535 promoter. Our future study will focus on transcriptional regulation of p53 in hsa-miR-4535 promoter. Furthermore, H_2_O_2_-induced collagen degradation could not be attenuated in hsa-miR-4535 overexpressed or si-Smad4 transfected cells even after galangin treatment (Figure 8C, 8E). These findings further confirm the involvement of hsa-miR-4535 in regulating collagen synthesis through targeting Smad4.

Accumulating evidences showed that UVB-induced ROS accumulation could impair skin integrity by altering ECM components as well as by decreasing collagen, elastin, and HA secretion and thereby facilitating wrinkle formation or skin cancer progression [61, 62]. Topical application of cosmetic products that contain natural compound to protect against sun burns could effectively alleviate UVB-induced damage [63]. Furthermore, dietary supplement of suberic acid protected SKH-1 hairless mice from UVB-induced collagen degradation and wrinkle formation [64]. In this study, we found that topical application of galangin attenuated UVB-induced skin photodamage by attenuating skin hyperplasia, wrinkle formation, and collagen synthesis (Figures 10B, 11, 12). However, galangin treatment did not significantly increased TGFβ/Smad signaling by comparing with UVB-exposed group in the *in vivo* study (Figure 12C). Total protein lysates from skin without separating dermis and epidermis layers for western blotting analysis were unable to detect the precise role of galangin in regulating TGFβ/Smad signaling. However, IHC staining of Smad 4 and p-smad2/3 and the amount of collagen (Figure 11) were enhanced in mouse dermis layers under galangin administration demonstrating the collagen promoting role of galangin. Taken together, these findings indicate that galangin could penetrate the epidermis and reach the dermis where it exerted its collagen producing abilities to suppress UVB-induced photodamage (Figure 13).

In conclusion, our findings indicated that galangin protects against H_2_O_2_/UVB-induced ROS generation, MMP imbalance, cellular senescence, and collagen degradation in human dermal fibroblasts. The dermo-protective effects of galangin were associated with the inhibition of hsa-miR-4535 and the activation of Smad2/3/4 complex to enhance collagen synthesis. Smad2/3/4 complex activation was regulated by hsa-miR-4535 through binding to Smad4-3′-UTR. Therefore, the protective mechanism of galangin against H_2_O_2_/UVB-induced skin dermal damage may involve the inhibition of Smad4/collagen synthesis by hsa-miR-4535.

## MATERIALS AND METHODS

### Cell cultures and treatments

HS68 human dermal fibroblasts were obtained from the Bioresource Collection and Research Center (BCRC, Hsinchu, Taiwan) and maintained in Dulbecco’s modified essential medium (DMEM, Thermo Fisher Scientific, MA, USA) containing 10% fetal bovine serum (Invitrogen, CA, USA), 2 mM L-glutamine (Sigma-Aldrich, MO, USA), 100 U/mL penicillin (Sigma-Aldrich), and 100 μg/mL streptomycin (Sigma-Aldrich) at 37°C and 5% CO_2_. To evaluate the effect of galangin against H_2_O_2_-induced cells damage, HS68 cells were treated with 200 μM of H_2_O_2_ for 1 h and then co-treated with galangin (282200, Sigma-Aldrich) for 23 h. Cells were washed with PBS and placed in 1 mL fresh PBS before UVB exposure. For UVB irradiation, the cells were exposed to a crosslinker (CX-2000, Upland, CA, USA) and UVB bulbs at an intensity of 40 mJ/cm^2^ and then treated with galangin.

### MTT assays

The HS68 cell viability after treatment was measured using MTT assay. HS68 cells were seeded into 96-well plates and treated with 200 μM H_2_O_2_ for 1 h or irradiated with 40 mJ/cm^2^ UVB and then incubated with different concentrations of galangin (10, 20, 30 μM) for 23 h. After 24 h, the culture medium was replaced with 100 μL MTT (3-(4,5-dimethylthiazol-2-yl)-2,5-diphenyl tetrazolium bromide) solution (0.5 mg/mL; 5 mg/mL stock solution in PBS was diluted with culture medium to 0.5 mg/mL) for 4 h at 37°C. Then the purple-colored formazan was solubilized in 100 μL of dimethyl sulfoxide (DMSO) and optical density at 570 nm was measured using a spectrophotometer (Bio-Rad, USA).

### Transients transfection

Plasmid encoding Smad4-3′-UTR in pmirGLO dual-luciferase miRNA target expression vector was purchased from GENEWIZ. Human siRNA Smad4 (ID-SASI_Hs01_00207793) and negative control siRNA (SIC001) were both purchased from Sigma-Aldrich. HS68 cells were transfected with hsa-miR-4535 inhibitor (anti-sense RNA), hsa-miR-4535 mimic (sense RNA), miRNA negative control, Smad4 siRNA using jetPRIME^®^ (Polyplus Transfection Inc, Illkirch, France) according to the manufacturer’s protocol. Then, the cells were incubated with the transfection complexes for 24 h.

### Microarray analysis

Total RNA was extracted from the control, 10 μM- and 30 μM-galangin-treated HS68 cell and then analyzed by miRNA Profiling using miRNA Microarray Services (Human miRNA OneArray^®^; Service Code: 1h216080404;).

### Luciferase reporter assay

Cells were co-transfected with a wild-type and mutant luciferase Smad4-3′-UTR-WT or Smad4-3′-UTR-MT reporter constructs, respectively, and internal control luciferase plasmids. After transfections, luciferase activities were detected by a Dual-Glo Luciferase Assay System (Promega, Sunnyvale, CA, USA) according to the manufacturer’s instructions. Plates were read on a Reporter Microplate Luminometer (Turner Biosystems, Sunnyvale, CA, USA). The relative luciferase activities were normalized to that of Renilla luciferase.

### Western blot analysis

Cells were washed using 1× phosphate-buffered saline (PBS) and lysed in lysis buffer (50 mM Tris-base (pH 7.5), 0.5 M NaCl, 1 mM EDTA (pH 8.0), 1 mM β-mercaptoethanol, 1% NP-40, 1% glycerol, and protease inhibitor cocktail tablets (Roche Molecular Biochemicals, Upper Bavaria, Germany) for 30 min on ice and then centrifuged at 12,000 × g for 10 min at 4°C. The supernatants were collected in 1.5 mL micro centrifuge tubes. The protein concentration in these supernatants was determined by Bradford method (Bio-Rad, Hercules, CA, USA). Samples containing equal amounts of protein (20 μg) were separated through 6%–12% gradient sodium dodecyl sulfate polyacrylamide gel electrophoresis (SDS-PAGE) and then transferred onto polyvinylidene difluoride (PVDF) membranes (Millipore, Bedford, MA, USA). Membranes were blocked using blocking buffer (5% non-fat dry milk, 20 mM Tris-HCl, pH 7.6, 150 mM NaCl, and 0.1% Tween 20) for 1 h at RT. Then, membranes incubated with primary antibodies (1:1,000 dilution) against TGFβ1 (sc-31609, Santa Cruz, CA, USA), p-smad2/3 (sc-11769, Santa Cruz), Smad4 (sc-7966, Santa Cruz), COL1A1 (sc-28657, Santa Cruz), COL3A1 (sc-271249, Santa Cruz), p16 (10883-1-AP, Proteintech), p21 (sc-6246, Santa Cruz), MMP-1 (sc-21731, Santa Cruz), GAPDH (sc-32233, Santa Cruz), and Histone H1 (sc-10806, Santa Cruz) overnight at 4°C. Then, they were incubated with appropriate secondary antibodies (1:80,000 dilution), antirabbit (A0545), antimouse (A9044), or antigoat (A5420) IgG (Santa Cruz Biotechnology) for 1 h at RT. The immunoblots were detected with enhanced chemiluminescence (ECL) horseradish peroxidase (HRP) substrate (Millipore) using ImageQuant LAS4000 mini (GE Healthcare Life Sciences, Little Chalfont, UK).

### Extraction of nuclear protein

Cytosolic and nuclear fractions were isolated using a Nuclear/Cytosol Fractionation Kit (BioVision, Milpitas, CA, USA) according to the manufacturer’s instructions. Briefly, cells were collected and lysed in cytosol extraction buffer (CEB) containing 1 mM DTT and protease inhibitors. After centrifugation at 12,000 rpm for 2 min, supernatants (cytosolic fractions) were transferred to new tubes. Then, nuclear extraction buffer (NEB) supplemented with 1 mM DTT and protease inhibitors was added, vortexed for 15 s, and incubated for 10 min for 40 min. Protein content was quantified by Bradford assay (Bio-Rad) and proteins were separated on 8%–12% gradient SDS-PAGE for western blot analysis.

### RNA extraction and qRT-PCR

Total RNA was extracted using a Direct-zol™ RNA MiniPrep commercial kit (Zymo Research Corporation, CA, USA) according to the manufacturer’s protocol [65, 66]. miRNAs were reverse transcribed to cDNA using a miRNA cDNA synthesis kit (Takara, Shiga, Japan) as described previously [16, 67]. hsa-miR-4535 and Smad4 expression was detected by real-time PCR in a LightCycler 96 Systems (Roche, Basel, Switzerland) using the standard LightCycler 480 SYBR Green I Master protocol. The 10 μL PCR reaction included 2 μL cDNA, 5 μL 2× SyberGreen PCR Mix, 0.5 μL forward primer (10 μM), 0.5 μL reverse primer (10 μM), and 2 μL ddH_2_O. All reactions were run in triplicate. For mRNA detection, threshold cycle (Ct) was determined for each gene and the expression of each gene relative to that of GAPDH was calculated as 2^ΔCt^ where ΔCt = (Ct_target gene_ – Ct_GAPDH_). For miRNA detection, Ct was determined for each gene and the expression of each gene relative to that of U6 rRNA was calculated as 2^ΔCt^ where ΔCt = (Ct_hsa-miR-4535_ – Ct_U6 rRNA_). The forward (F) and reverse (R) primers used to detect miRNA and mRNA expression were: hsa-miR-4535 (3′-CAGGGTCGGUCCA5′); human-Smad4 (F′-TGACCCAAACATCACCTTCA, R′-GATACGTGGACCCTTCTGGA); human-GAPDH (F′-ACCCAGAAGACTGTGGATGG, R′-TTCAGCTCAGGGATGACCTT); mouse-Smad4 (′’-GTGGAAGCCACAGGAATGTT, R′-CCCACTGAAGGACATTCGAT); mouse-GAPDH (F′-CCTTCCACAATGCCAAAGTT, R′-GGGTGTGAACCACGAGAAAT).

### Immunofluorescence analysis

HS68 cells were fixed using 4% paraformaldehyde for 15 min at RT, permeabilized with 0.1% Triton X-100 for 15 min at RT, and then blocked with 5% bovine serum albumin for 10 min at RT. Thereafter, the cells were washed and stained with Alexa Fluor 594 goat anti-mouse IgG or Alexa Fluor 488 donkey anti-goat IgG secondary antibodies (Invitrogen, Carlsbad, CA, USA) for 2 h at RT. Next, cells were washed with 1× PBS and stained with DAPI to detect cell nucleus (blue stain) for 1 min at RT. Images for Smad4 and p-smad2/3 staining in HS68 cells were captured using a fluorescence microscope (DP73, Olympus, Tokyo, Japan).

### Detection of total and mitochondrial ROS

Total and mitochondrial ROS were detected using 2′,7′-Dichlorofluorescin diacetate DCFH-DA (D6883, Sigma-Aldrich) and MitoSOX red mitochondrial superoxide indicator (Invitrogen, M36008). HS68 Cells were incubated with 5 μM DCFH-DA and 2 μM MitoSOX at 37°C for 30 min. Then, the cells were washed with 1× PBS and stained with DAPI to detect cell nucleus (blue stain) for 1 min at RT. All samples were analyzed using a fluorescence microscope (DP73, Olympus).

### Measurement of mitochondrial membrane potential (MMP)

MMP was determined by detecting the changes in mitochondrial potential using a mitochondria staining kit (CS0390, Sigma-Aldrich) according to the manufacturer’s instructions. HS68 cells were seeded in 4-well slides for 24 h before treatment. After treatment, cells were washed by 1× PBS and incubated with JC-1 at 37°C for 20 min and then washed twice with JC-1 dyeing buffer. Images were captured using a fluorescence microscope (Olympus). Red fluorescence (aggregated JC-1) indicated intact mitochondria, while green fluorescence (monomeric JC-1) indicated impaired mitochondria.

### Annexin V and propidium iodide (PI) staining by flow cytometry

Apoptotic cells were stained with Annexin V and PI and then detected by flow cytometry using staining. Briefly, HS68 cells were treated with different concentrations (50, 100, 200, 300, and 400 μM) of H_2_O_2_ for 24 h. The cells were then collected by trypsinization and stained using an Annexin V and PI apoptosis detection kit (BD Biosciences, San Jose, CA, USA) according to the manufacturer’s protocol. Flow cytometry was performed at the Fluorescence Activated Cell Sorting (FACS) Core Facility, China Medical University, Taiwan, using a FACS Canto™ system (BD Biosciences).

### Senescence-associated β-galactosidase (SA-β-gal) staining

SA-β-gal staining kit (9860, Cell Signaling, Danvers, MA, USA) was used to detect senescent cells. Briefly, HS68 cells (20,000 cells/well) were cultured on slides for 24 h, fixed with 4% paraformaldehyde for 10 min, washed with ice-cold PBS, and then incubated overnight at 37°C with X-Gal at pH 6.0. After washing, the blue-stained aging cells were photographed using a light microscope (Olympus, Tokyo, Japan).

### Animal models

Four-weeks-old C57BL/6J nude mice were obtained from BioLasco Taiwan Co., Ltd, Taipei, Taiwan. In accordance with the National Laboratory Animal Center (NLAC) protocols, animals were housed at China Medical University Animal Center under controlled temperature conditions (22–24°C) and 12-h light-dark cycle with free access to standard laboratory diet and tap water during the experimental period. The dorsal skin regions of mice were marked with India ink and the animals were divided into four groups (*n* = 6/group): control plus vehicle group, UVB-irradiated plus vehicle group, UVB-irradiated plus low-dose (12 mg/kg) galangin-treated group, and UVB-irradiated plus high-dose (24 mg/kg) galangin-treated group. The allocated skin areas were irradiated with 150 mJ/cm^2^ UVB per exposure and topically treated with vehicle or galangin three times a week for 8 weeks. After weighing mouse body weight, galangin was freshly prepared at a concentration of 12 mg/kg and 24 mg/kg in propylene glycol/ethanol/H_2_O (1:1:8). Solution (100uL) was topically applied to the designated sites (2 × 2 cm) on dorsal skin of mice. For UVB exposure, mice were placed in a UV crosslinker box which was equipped with UVB bulbs. Before mice received UVB irradiation, control (non UVB-exposed area) was covered with anti-UV sticker. Different doses of galangin or vehicle were topically applied on the dorsal skin of mice after UVB exposure. At the end of the experiment all animals were weighed and sacrificed. Then, their dorsal skin was quickly excised, immediately frozen in liquid nitrogen, and stored at −80°C for protein and RNA analyses; a part was fixed with 10% formalin solution for histological staining.

### Histological examination

Skin biopsy specimens were fixed in 10% formalin and rehydrated using an alcohol gradient (100%, 95%, and 75%) for at least 24 h. Then, the samples were embedded in paraffin wax and sectioned at 0.5 μm thickness. The embedded skin slices were stained with hematoxylin and eosin (H&E) and Masson's trichrome (MT) to assess the skin structure and collagen fiber content, respectively. Images were captured using a light microscope (Olympus).

### Immunohistochemistry

The embedded 0.5 μm-thick skin tissue samples was dried at 60°C overnight following dewaxing in xylene for 30 min, and rehydration in 100% ethanol for 30 min. Endogenous peroxidase activity was blocked by incubation in hydrogen peroxide blocking buffer (3% H_2_O_2_) for 10 min. After rinsing the sections with tap water for 15 min, nonspecific binding was blocked by incubation with 2.5% horse serum for 10 min. Then, the sections were incubated with primary antibodies (1:100 dilution) overnight at 4 °C. After washing with 1× PBS, the samples were treated with secondary antibody for 10 min at RT. Subsequently, HRP Polymer was added to the slides for 10 min at RT, followed by DAB substrate (Roche) application for 15 s at RT. Then, the sample was stained with hematoxylin for 5 min at RT. Finally, the tissue–polymer complex was detected using a light microscope (Olympus).

### Statistical analysis

All experiments were performed at least three times. Statistical analyses were performed using Graph Pad Prism5 statistical software (Graph Pad Software Inc., San Diego, CA, USA). Differences between the means of multiple groups were assessed by analysis of variance. Data were analyzed by separate one-way ANOVA. Differences between individual means were determined by either Tukey’s or Dunnett’s tests. Quantitative data are presented as the mean ± SD corresponding of three or more replicates. A *P*-value < 0.05 was considered statistically significant.
